# Astroglial CD38 impairs hippocampal synaptic plasticity after global cerebral ischemia

**DOI:** 10.3389/fstro.2024.1423887

**Published:** 2024-08-14

**Authors:** Amelia M. Burch, Ami Haas, James E. Orfila, Erika Tiemeier, Cassidy De Anda Gamboa, Nicholas Chalmers, Nidia Quillinan, Paco S. Herson

**Affiliations:** ^1^Department of Anesthesiology, Neuronal Injury & Plasticity Program, University of Colorado School of Medicine, Aurora, CO, United States; ^2^Department of Neurological Surgery, The Ohio State University College of Medicine, Columbus, OH, United States

**Keywords:** CD38, cardiac arrest, global cerebral ischemia, TRPM2, hippocampus, LTP

## Abstract

Cardiac arrest-induced global cerebral ischemia (GCI) results in profound cognitive impairment in survivors. Our prior work demonstrated persistent disruption of long-term potentiation (LTP) in hippocampal CA1 neurons, correlating with learning and memory deficits in a rodent model of cardiac arrest/cardiopulmonary resuscitation (CA/CPR). Delayed inhibition of the Ca^2+^-permeable TRPM2 ion channel restored LTP post-CA/CPR, yet the mechanisms upstream of TRPM2 activation remain elusive. This study investigates CD38 as a potential regulator of TRPM2, highlighting a novel target to reverse hippocampal synaptic plasticity deficits after ischemia. We observe elevated levels of CD38 in activated astrocytes in the CA1 region of the hippocampus 7 days following CA/CPR in both male and female mice. Delayed inhibition of CD38 reverses hippocampal synaptic plasticity impairments at subacute timepoints after CA/CPR, phenocopying TRPM2 restoration of LTP. Our previous findings demonstrated that TRPM2 inhibition reverses the CA/CPR-induced enhancement of GABA_A_ receptor (GABA_A_R) clustering, which contribute to ongoing LTP deficits. We, therefore, assessed the effect of CD38 on GABAergic inhibitory potentiation and find that inhibition of CD38 reverses GABA_A_R clustering in a TRPM2-dependent manner. In this study, we identify astroglial CD38 as a potential target and upstream regulator of the TRPM2 channel, offering a promising approach to restore hippocampal synaptic plasticity impairments following GCI through modulation of GABAergic signaling.

## Graphical Abstract

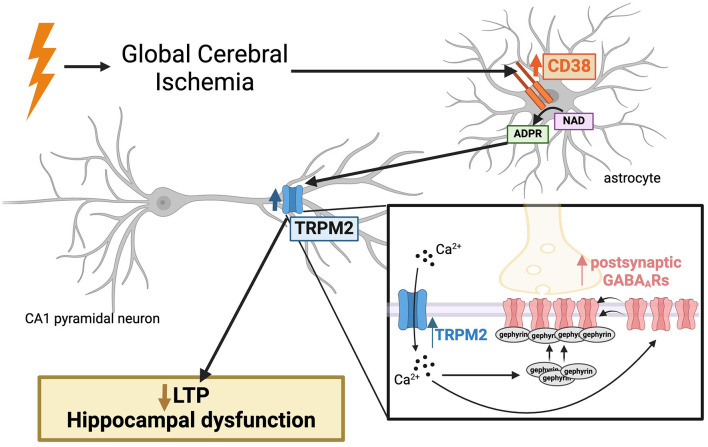

## Introduction

Cardiac arrest leads to global cerebral ischemia (GCI), causing compromised hippocampal function and lasting cognitive impairment in survivors (Petito et al., 1987; Moulaert et al., 2009; Sabedra et al., 2015). Traditional neuroprotective pharmacotherapies targeting acute neuronal cell death have shown limited efficacy in providing long-term functional benefits to survivors (Cheng et al., 2004; Wahlgren and Ahmed, 2004; Katz et al., 2022), creating a need for alternative therapies to improve functional outcomes. Neurorestoration is an alternative approach which aims to intervene within a broader therapeutic timeframe by restoring disrupted neuronal circuits and synaptic function (Azad et al., 2016; Escobar et al., 2019); however, potential targets for drug development remain scarce.

Using a murine model of cardiac arrest/cardiopulmonary resuscitation (CA/CPR), we previously demonstrated prolonged deficits in hippocampal synaptic plasticity and learning and memory for at least 30 days following GCI, a timepoint at which cell death processes are largely complete (Orfila et al., 2014; Dietz et al., 2020). Remarkably, delayed inhibition of the Ca^2+^-permeable transient receptor potential melastatin-2 (TRPM2) ion channel reversed CA/CPR-induced impairments in hippocampal long-term potentiation (LTP) and learning and memory in both sexes for at least 30 days after the injury (Dietz et al., 2020, 2021). Despite this data implicating TRPM2 as a promising candidate for neurorestorative therapy, the precise upstream mechanisms governing ongoing TRPM2 activation in both sexes after ischemia are largely unknown.

CD38 is an ectoenzyme which converts NAD to ADP-ribose, the TRPM2 ion channel ligand (Malavasi et al., 2008). While CD38 is primarily known for its role in NAD depletion in aging and tumorigenesis (Chatterjee et al., 2018; Tarrago et al., 2018; Hogan et al., 2019; Chini et al., 2020), several studies have also highlighted its relevance in brain ischemia. Genetic depletion of CD38 is neuroprotective (Long et al., 2017) and attenuates chemokine production and the neuroimmune response after focal cerebral ischemia (Choe et al., 2011). While broadly expressed in immune cells, CD38 upregulation in activated astrocytes has been observed in numerous neuroinflammatory states (Piedra-Quintero et al., 2020). Reactive astrogliosis is a prominent feature of CNS repair, observed several days after the initial ischemic insult (Pekny and Nilsson, 2005). While activated astrocytes acutely limit tissue damage following insults, protracted astrogliosis has been shown to negatively impact functional recovery and neuroplasticity processes (Pekny et al., 2014, 2019). Therefore, while CD38 has been implicated in the acute cell death response after ischemia, its potential role in hippocampal synaptic plasticity deficits in the subacute/chronic phase remains unclear, despite its established expression in activated astrocytes and its generation of ADP-ribose, a the TRPM2 ion channel ligand.

Maintaining a balanced excitatory/inhibitory (E/I) ratio is crucial for normal brain function and synaptic plasticity (Smith and Kittler, 2010; Vogels et al., 2011). GABAergic inhibitory synapses play an essential role in governing excitatory synaptic plasticity, particularly LTP (Steele and Mauk, 1999; Leao et al., 2012; Williams and Holtmaat, 2019; Udakis et al., 2020). Clustering of GABA_A_ receptors (GABA_A_Rs) within the postsynaptic membrane is one mechanism by which inhibitory synapses are fine-tuned and may regulate excitatory synaptic plasticity (Nusser et al., 1997; Luscher et al., 2011). We previously reported its relevance following GCI, showing enhanced GABA_A_R clustering and function contributing to the persistent LTP deficits and E/I imbalance after CA/CPR (Burch et al., 2024). Moreover, we demonstrated GABAergic synaptic potentiation required a TRPM2-dependent pathway (Burch et al., 2024). Given the known relevance of CD38 in ischemic injury and its synthesis of the TRPM2 ligand, ADP-ribose, we tested the hypothesis that CD38 is upregulated after ischemic injury, contributing to hippocampal synaptic plasticity deficits through TRPM2-mediated enhancement of GABAergic function.

Here, we demonstrate an increase in CD38 protein and mRNA levels in activated astrocytes 7 days following GCI in both male and female mice. We find GCI-induced LTP impairments are reversed following delayed inhibition of CD38 in both sexes, mimicking the reversal of LTP deficits after TRPM2 blockade (Dietz et al., 2020, 2021). Furthermore, we observe a TRPM2-dependent restoration in the clustering and density of GABAergic synaptic proteins after CD38 blockade following oxygen-glucose deprivation (OGD). In summary, our evidence indicates enhanced expression of astroglial CD38 contributes to ongoing hippocampal synaptic plasticity deficits after GCI by augmenting GABA_A_R clustering via TRPM2 activation, presenting a novel target for pharmacotherapy.

## Materials and methods

### Experimental model and subject details

#### Animals

All studies conformed to the requirements of the National Institutes of Health *Guide for the Care and Use of Laboratory Animals* and were approved by the Institutional Animal Care and Use subcommittee of the University of Colorado, Denver AMC. C57BL/6 mice were bred in house in the Animal Resource Center at the University of Colorado Anschutz Medical Campus and monitored regularly for health. Mice were weaned between postnatal day 21 and 28 (P21–28) and housed in micro-isolator cages on a 14:10 light: dark cycle with water and chow available *ad libitum*.

#### Cardiac arrest/cardiopulmonary resuscitation

Female and male mice that were approximately 8–12 weeks old were subjected to either cardiac arrest and cardio-pulmonary resuscitation (CA/CPR) or sham procedures as described previously (Deng et al., 2017; Dietz et al., 2020). Briefly, mice were anesthetized with 3% isoflurane. Mice were intubated and connected to a mouse ventilator set to 160 breaths per min. Cardiac function was monitored via electrocardiography, and pericranial temperature was maintained at 37.5°C ± 0.2°C using a water-filled coil. Asystolic cardiac arrest was induced by KCl injection via jugular catheter. CPR begun 6 min after induction of cardiac arrest, by slow injection of 0.5–1.0 mL of epinephrine (16 μg epinephrine/mL, 0.9% saline), chest compressions at a rate of ~300 min^−1^, and ventilation with 100% oxygen. If return of spontaneous circulation could not be achieved within 3 min of CPR, resuscitation was terminated and the mouse was excluded from the study. After surgical procedures, mice were housed individually in micro-isolator cages on heating pads. Post-surgical care included daily saline injections (1 mL) and moist chow for 72 hrs. Investigators performed all experiments blind to surgical procedure of the animal, with separate investigator generating the code.

#### Dissociated hippocampal cultures

Primary hippocampal cultures were prepared as described previously (Crosby et al., 2019; Rajgor et al., 2020; Garcia et al., 2021). Briefly, the hippocampi from neonatal rat pups (P0-P1) were dissected and dissociated in papain. The isolated neurons were then seeded in MEM supplemented with 10% FBS and penicillin/streptomycin. Cells were plated at a density of 150,000–200,000 cells per 18 mm, #1.5 glass coverslip coated with poly-D-lysine. The MEM was replaced with Neurobasal (NB) media (GIBCO) supplemented with B27 (GIBCO) and 2 mM Glutamax 24 h after plating. The media were refreshed every 5 days be removing half of the existing media and replacing it with fresh NB media. To restrict the growth of actively dividing cells, mitotic inhibitors (uridine fluoro deoxyuridine) were introduced on day 5. The cultures were maintained at 37°C with 5% CO_2_ for a period of 13–14 days before conducting OGD experiments.

### Method details

#### Hippocampal slice preparation

Hippocampal slices were prepared 7 days post-surgical procedures. Mice were anesthetized with 3% isoflurane in an oxygen enriched chamber then trans-cardially perfused with oxygenated ice-cold artificial Cerebral Spinal Fluid (aCSF) containing in mM: 126 NaCl, 25 NaHCO_3_, 12 glucose, 2.5 KCl, 2.4 CaCl_2_, 1.3 NaHPO_4_, and 1.2 MgCl_2_. Horizontal slices (300 μM) were cut in aCSF supplemented with 9 mM MgSO_4_ and continuous oxygenation using a Vibratome 1,200 (Leica) and transferred to a holding chamber containing aCSF warmed to 33°C. After 30 min, slices recovered for an additional 30 min at room temperature (RT), prior to electrophysiology recordings.

#### Field electrophysiology

Hippocampal slices were placed in a heat-controlled interface chamber perfused with aCSF at a rate of 1.5 mL/min at 32°C. Responses were evoked using an insulated tungsten bipolar stimulating electrode placed in stratum radiatum to stimulate Schaffer collateral-commissurals (SCC), and recorded with a glass electrode containing 150 mM NaCl placed in the distal dendrites of CA1 pyramidal cell layer. Analog field excitatory postsynaptic potentials (fEPSPs) were amplified (1,000 × ) and filtered through a preamplifier (Grass Model P511) 0.03 Hz to 1.0 kHz, digitized at 10 kHz and stored on a computer for later off-line analysis (Datawave Technologies). The derivative (dV/dT) of the initial fEPSP slope was measured. The fEPSPs were adjusted to 50% of the maximum slope and test pulses were evoked every 20s. Paired pulse responses were recorded using a 50-ms interpulse interval (20 Hz) and expressed as a ratio of the slopes of the second pulse over the first pulse. Following the baseline recording, theta burst stimulation (TBS) was delivered, which included a train of four pulses delivered at 100 Hz in 30-ms bursts repeated 10 times with 200-ms interburst intervals. Following TBS, the fEPSP was recorded for 60 min. The averaged 10-min slope from 50 to 60 min after TBS was divided by the average of the 10-min baseline (set to 100%) prior to TBS to determine the amount of potentiation. For time course graphs, normalized fEPSP slope values were averaged and plotted as the percent change from baseline. For conditions requiring 78c treatment, 100 nM of the inhibitor was added to the aCSF and bath applied to the slice for 20 min prior to baseline recording and 78c was continuously bath applied throughout the experiment. 78c is a selective and potent CD38 inhibitor with a K_i_ in the low nanomolar range (Tarrago et al., 2018).

#### Immunohistochemistry

Mice were anesthetized and transcardially perfused with ice-cold PBS followed by 4% paraformaldehyde (PFA). Whole brains were removed and post-fixed in 4% PFA at 4°C overnight. After 24 h, brains were transferred to a glycerol and Sorenson's Buffer cryoprotection solution for long term storage. Frozen coronal sections were made using a sliding microtome, and slices placed in cryostorage solution containing phosphate buffer, ethylene glycol, polyvinylpyrolidine, and sucrose and stored at 4°C until staining was performed. Free floating sections were washed for 15 min (3X) in PBS at RT then blocked and permeabilized (5% BSA, 5% NGS, 0.5% Triton X-100 and 1X PBS) at RT for 5–6 h on a rocker. Slices were incubated with CD38 (1:30 Santa Cruz Mouse- 147011) and GFAP (1:5,000 Wako Rabbit – GA524) antibodies in permeabilization solution overnight at 4°C on a rocker. Slices are washed for 20 min in PBS (4X) and incubated with appropriate secondary antibodies (1:1,000 ThermoFisher, Alexa-Fluor 488 and 594) for 1 h in blocking solution. Prior to mounting with ProLong Gold, slices were washed for 20 min in PBS (4X).

#### Fluorescence in situ hybridization

To detect *cd38* and *gfap* mRNA from fresh frozen hippocampal sections, fluorescent *in situ* hybridization (FISH) was performed using RNAscope^®^ Multiplex Fluorescent Reagent Kit (cat# 320293, Advanced Cell Diagnostics) according to the protocol provided by the manufacturer. Briefly, microtome-cut 50 μM frozen sections were mounted on charged slides, air dried, and fixed in 4% paraformaldehyde buffer. Following fixation, sections underwent a series of ethanol dehydration steps (50%, 70%, 80%, and 100%) and were heated in manufacturer-provided antigen retrieval buffer. Sections were then subjected to protease (kit-provided) digestion for 30 minutes at 40°C. For hybridization, sections were exposed to probes (C1 *cd38* cat #513061, C2 *gfap* cat #313211-C2, Advanced Cell Diagnostics) and incubated at 40°C in a hybridization oven for 2 h. Following washes, sections underwent signal amplification steps and fluorophore (1:750 dilutions; Opal™ Dye 520 cat# FP1487001KT, Opal™ Dye 570 cat# 1488001KT; Akoya Biosciences) treatments according to manufacturer's instructions. Sections were counterstained with DAPI (kit-provided), stored at 4°C and imaged within 2 weeks. Images were acquired at 40 × from the hippocampal CA1 region in the ipsilateral and contralateral hemispheres from two young adult CA/CPR male and female mice.

#### Immunocytochemistry

Coverslips containing neuronal cultures were fixed in a 4% PFA solution consisting of 4% sucrose, 1X PBS, and 50 mM HEPES (pH 7.4) for 5 min at RT. After fixation, the cells were blocked in a solution containing 5% BSA, 2% Normal Goat Serum (NGS), and 1X PBS at RT for 30 min. Staining for surface GABA_A_R-γ2 subunit (1:500, Synaptic Systems, guinea pig-224004) was performed under non-permeabilized conditions in the blocking solution for 1 h at RT. Following the primary antibody incubation, coverslips were washed three times for 5 min each with 1X PBS. Subsequently, permeabilization was carried out using 0.5% NP-40 for 2 min, followed by blocking at RT for 30 min. Staining for gephyrin (1:600, Synaptic Systems, mouse, 3B11 clone, 147111) and VGAT (1:1000, Synaptic Systems, rabbit, 131003) was performed in the blocking solution for 1 h, following by three 5 min washes with PBS. The coverslips were then incubated with appropriate secondary antibodies (1:1,000, ThermoFisher, Alexa-Fluor 488, 568, and 647). Coverslips were washed three times for 5 min and were then mounted on microscope slides using ProLong Gold mounting media (ThermoFisher).

#### Oxygen glucose deprivation in neuronal culture

OGD was induced in DIV13-15 hippocampal neuronal cultures using HEPES-buffered solution. The OGD-HEPES solution contained (in mM): 25 HEPES (pH 7.4), 140 NaCl, 5 KCl, 2 CaCl_2_, 1 MgCl_2_ and 10 sucrose (or supplemented with 10 mM glucose for control conditions). Prior to OGD treatment, the OGD-HEPES solution was placed in an anaerobic workstation at 37°C with a controlled atmosphere of 95% N_2_ and 5% CO_2_ (Bugbox Plus, Baker Co) for 24 h to allow for deoxygenation. Neuronal cultures were washed twice and incubated with OGD-HEPES solution in the anoxic chamber for 20 min. Reperfusion was then initiated by replacing the OGD-HEPES solution with glucose-containing conditioned media and returning coverslips to an aerobic incubator. After 96 h in aerobic conditions, coverslips were fixed for immunocytochemistry. For the treatment conditions, coverslips were treated with 100 nM 78c or combined with 2μM tatM2NX for 1 h prior to fixation. Control neurons were incubated at 37°C, 5% CO_2_ with Control-HEPES solution for 20 min and returned to conditioned media before fixation at the 96 h timepoint.

### Image acquisition and data analysis

#### Confocal microscopy

Cultured pyramidal neurons were imaged using an Olympus FV1000 laser scanning confocal microscope, 60 × oil immersion objective with 2 × digital zoom and Fluoview software (Olympus FluoView, FV10-ASW). Images were attained at 0.3 μm intervals (4 μm Z-stack projection). Cluster analysis was performed using ImageJ (NIH) by selecting regions of interest (ROIs) to delineate dendritic compartments. A user-based threshold was determined by sampling several images per condition across all conditions and clusters were defined as a minimum size of 0.05 μm^2^. Density of clusters was calculated by measuring the number of clusters divided by the length of the delineated dendrites (per 10 μm). A total of 30–36 neurons were analyzed per condition from three independent hippocampal preparations.

For IHC experiments, CA1 hippocampus was identified by DAPI staining of the pyramidal cell layer. ROIs captured both the pyramidal cell layer and *stratum radiatum* in the same frame, and different user-based thresholds were used for the cell bodies and dendrites. A user-based threshold was determined by sampling several images per condition across all conditions. Percent positive pixels were then measured using this threshold. Pearson's correlation coefficient was calculated to assess CD38 colocalization to GFAP using the JACoP plugin in ImageJ. A minimum of 6 animals per condition were utilized for IHC. Analysis was performed blind to surgical condition.

#### Experimental design and statistical analysis

All analyses were conducted blind to condition. Number of animals and cells are indicated in the figure legend. All data in figures are presented as mean ± SEM. Statistical significance was determined using appropriate tests indicated in the figure legends. Normality confirmed with Shapiro-Wilks. A *p*-value ≤ 0.05 was used to declare significance. All statistical analyses were performed on GraphPad Prism v9.4.

## Results

### CD38 is upregulated in activated astrocytes following CA/CPR

**Figure 1:**
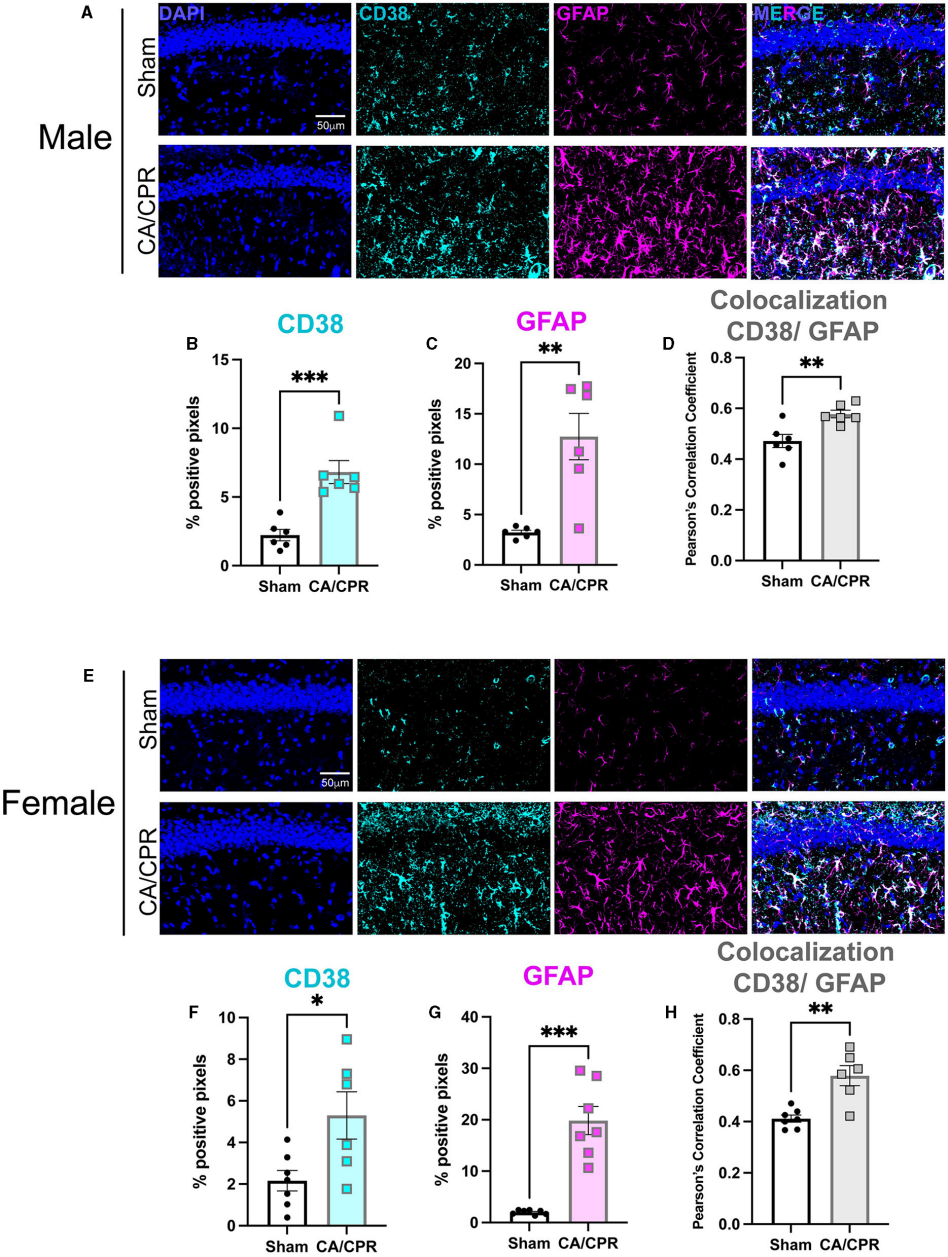
CD38 is upregulated in activated astrocytes following CA/CPR. **(A)** Representative confocal images of DAPI (blue), CD38 (cyan) and GFAP (magenta)immunohistochemical staining from CA1 hippocampal sections of 7-day sham andCA/CPR male mice. **(B, C)** Quantification of percent positive pixels of **(B)** CD38 and **(C)** GFAP staining in male mice; *n* = 6 animals per condition; unpaired *t*-test. **(D)** Quantification of CD38 colocalization to GFAP using Pearson's Correlation Coefficient in malemice; *n* = 6 animals per condition; unpaired *t-*test. **(E)** Representative confocal images of DAPI (blue), CD38 (cyan) and GFAP (magenta)immunohistochemical staining from CA1 hippocampal sections of 7-day sham andCA/CPR female mice. **(F, G)** Quantification of percent positive pixels of **(B)** CD38 and **(C)** GFAP staining in female mice; *n* = 6–7 animals per condition, unpaired *t*-test. **(H)** Quantification of CD38 colocalization to GFAP using Pearson's Correlation Coefficient infemale mice *n* = 6–7 animals per condition, unpaired *t*-test. Values represent mean ± SEM. *p < 0.05; **p < 0.01, ***p < 0.001.

To assess the expression and localization of CD38 after GCI, we employed immunohistochemistry (IHC) and stained for CD38 and GFAP, the activated astrocyte marker, in CA1 hippocampal sections 7 days following CA/CPR or sham surgery in both male (Figure 1A) and female (Figure 1E) mice. More CD38 staining was observed in CA/CPR compared to sham as measured by percent positive pixels (Sham: 2.228% ± 0.4104% vs. CA/CPR: 6.823% **±** 0.8380%, *p* = 0.006; Figure 1B), indicating an elevation in protein levels of CD38 after GCI. We also found an increase in GFAP staining following CA/CPR (Sham: 3.227% ± 0.2149% vs. CA/CPR: 12.74% ± 2.303%, *p* = 0.0021; Figure 1C), suggesting astrocytes remain persistently activated at subacute timepoints after ischemia. Further, the increase in Pearson's correlation coefficient measuring CD38 overlap with GFAP suggested a higher degree of CD38 colocalization to astrocytes after GCI (Sham: 0.4717 ± 0.02617 vs. CA/CPR: 0.5780 ± 0.01508, *p* = 0.0055; Figure 1D). In females, we observed similar changes, finding both an increase in percent positive pixels in CD38 (Sham: 2.160% ± 0.4911% vs. CA/CPR: 5.301% ± 1.140%, *p* = 0.0217; Figure 1F) and GFAP (Sham: 2.032% ± 0.1632% vs. CA/CPR: 21.37% ± 2.673%, *p* < 0.0001; Figure 1G) staining and an increase in CD38 colocalization to GFAP 7 days after CA/CPR (Sham: 0.4117 ± 0.01422 vs. CA/CPR: 0.5793 ± 0.03915, *p* = 0.0013; Figure 1H). CD38 has also been identified in microglial cell types (Mayo et al., 2008; Hannawi et al., 2022). We, therefore, employed immunohistochemical staining again to assess its colocalization with the microglial marker, Iba1 in both male (Supplementary Figure S1A) and female (Supplementary Figure S1D) mice. Males exhibited no difference in Iba1 colocalization after CA/CPR (Sham: 0.2043 ± 0.02490 vs. CA/CPR: 0.1963 ± 0.0378, *p* = 0.8690; Supplementary Figure S1B). CD38 colocalization was also significantly higher when co-stained with GFAP compared to Iba1 in males after CA/CPR (Iba1: 0.1963 ± 0.0378 vs. GFAP: 0.5780 ± 0.01508, *p* < 0.0001; Supplementary Figure S1C). We observed a similar effect in females, with a decrease in CD38 colocalization to Iba1 after CA/CPR (Sham: 0.2093 ± 0.01626 vs. CA/CPR: 0.1396 ± 0.02151, *p* = 0.0288; Supplementary Figure S1E) and a significantly greater CD38 overlap with the GFAP stain compared to Iba1 (Iba1: 0.1396 ± 0.02151 vs. GFAP: 0.5793 ± 0.03915, *p* < 0.0001; Supplementary Figure S1F). Taken together, we found CD38 is upregulated in astrocytes and not in microglia at subacute timepoints following cardiac arrest, with no evidence of sexual dimorphism.

### CD38 mRNA levels appear to be increased and localized to activated astrocytes following CA/CPR

**Figure 2:**
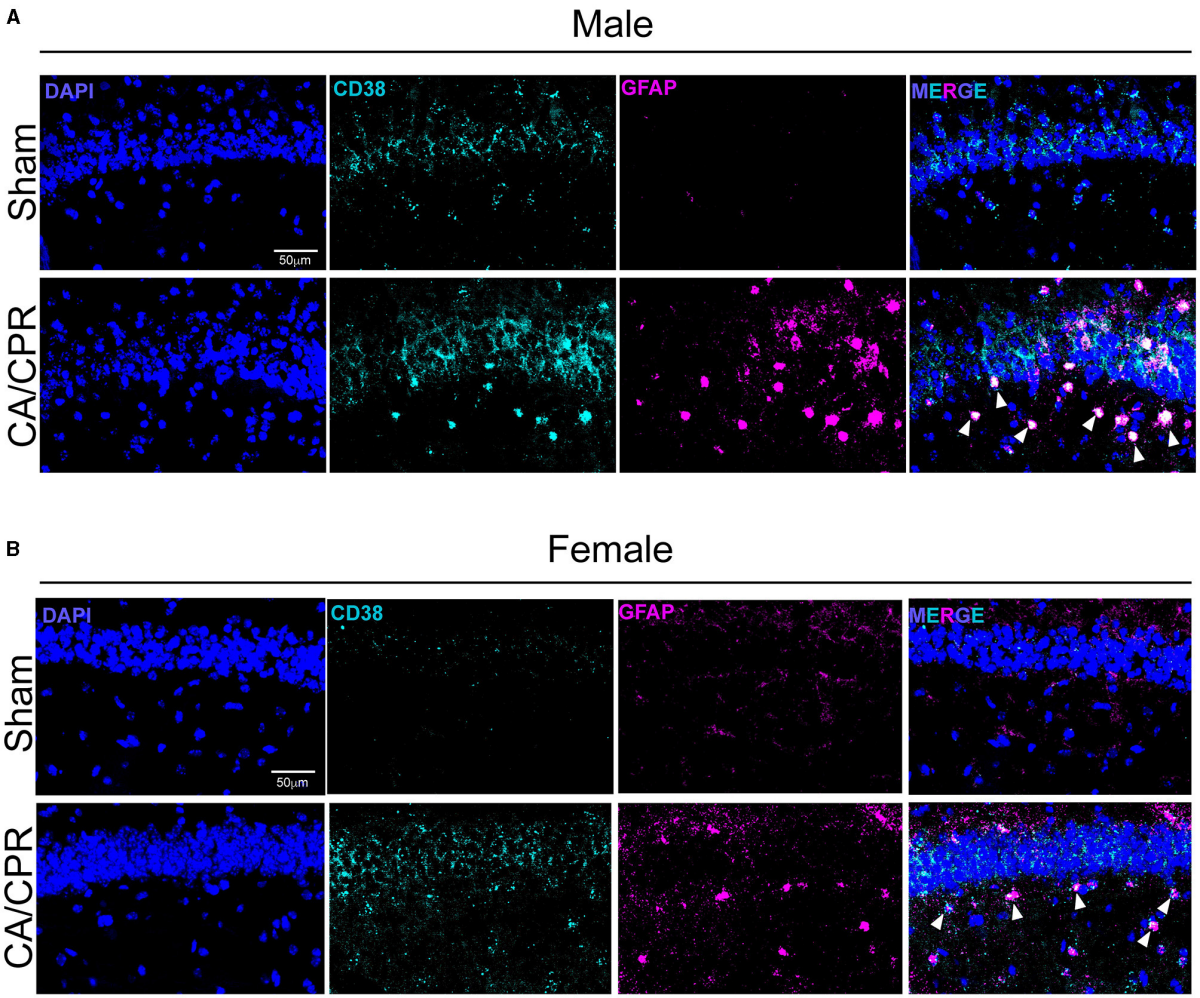
CD38 mRNA levels appear to be increased and localized to activated astrocytes following CA/CPR. **(A)** Representative confocal images of fluorescence *in situ* hybridization using probes directedagainst *cd38* (cyan) and *gfap* (magenta) mRNA transcripts in hippocampal sections of 7-day sham **(top)** and CA/CPR **(bottom)** male mice. Sections were counterstained with DAPI(blue) to delineate the CA1-hippocampal region. **(B)** Representative confocal images of fluorescence *in situ* hybridization using probes directedagainst *cd38* (cyan) and *gfap* (magenta) mRNA transcripts in hippocampal sections of 7-day sham **(top)** and CA/CPR **(bottom)** female mice. Sections were counterstained withDAPI (blue) to delineate the CA1-hippocampal region.

To verify the results obtained by IHC, we performed fluorescence *in situ* hybridization (FISH) to qualitatively assess CD38 mRNA transcript levels and localization. We probed for *gfap* and *cd38* transcripts in the CA1 region of the hippocampus 7 days following sham and CA/CPR surgeries. In males, we found an apparent increase in *cd38* and *gfap* mRNA, with strong colocalization of both transcripts (Figure 2A), consistent with the IHC data shown in Figure 1. We obtained similar results in female mice, with an obvious increase in both transcripts and colocalization (Figure 2B). Both the IHC and the FISH data strongly suggest an upregulation of CD38 in astrocytes at subacute timepoints following CA/CPR.

### Delayed inhibition of CD38 restores hippocampal long-term potentiation after CA/CPR

**Figure 3:**
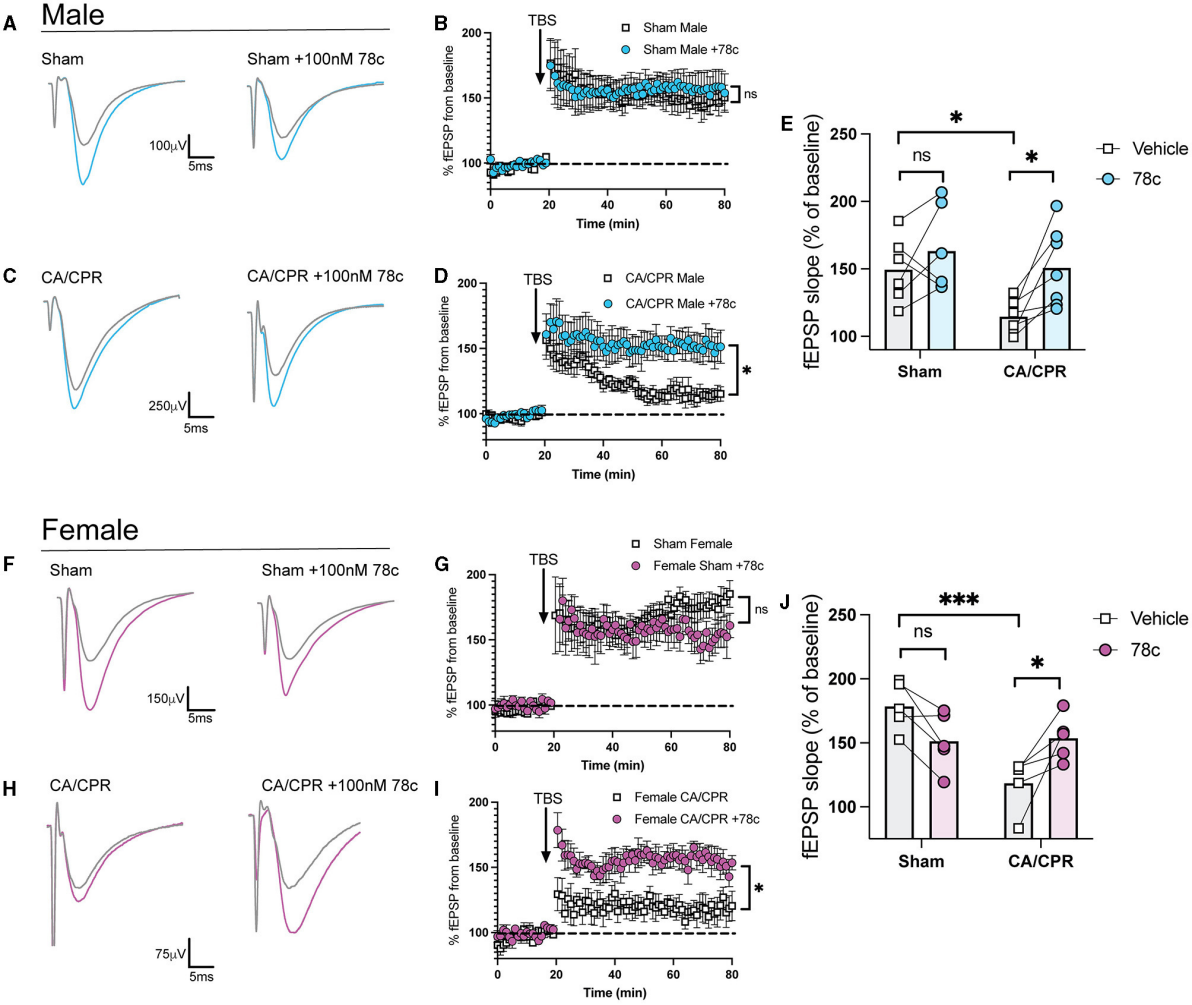
Delayed inhibition of CD38 restores hippocampal long-term potentiation after CA/CPR. **(A)** Representative traces from sham male mice of field excitatory postsynaptic potentials (fEPSPs) before theta burst stimulation (TBS) (gray) and after TBS (blue). LTP experiments wereperformed in the same animal in the absence **(left)** and presence of 78c (100 nM) **(right)**. **(B)** Time course graph measuring fEPSPs from sham male mice normalized to the average of thelast 10 min of baseline in the absence (white) or presence (blue) of 78c (100 nM). **(C)** Representative traces from CA/CPR male mice of field excitatory postsynaptic potentials (fEPSPs) before theta burst stimulation (TBS) (gray) and after TBS (blue). LTP experiments were performed in the same animal in the absence **(left)** and presence of 78c (100 nM) **(right)**. **(D)** LTP data measuring fEPSPs from CA/CPR male mice normalized as a percentage of baselinein the absence (white) or presence (blue) of 78c (100 nM) in different sections from thesame animal. **(E)** Normalized slope of fEPSPs after theta burst stimulus in male mice; *n* = 6–7 animals percondition; Two-Way ANOVA with Repeated Measures, Sidak's *post hoc* test. **(F)** Representative traces from sham female mice of field excitatory postsynaptic potentials (fEPSPs)beforetheta burst stimulation (TBS) (gray) and after TBS (pink). LTP experiments wereperformed in the same animal in the absence **(left)** and presence of 78c (100 nM) **(right)**. **(G)** Time course graph measuring fEPSPs from sham female mice normalized to the average of thelast 10 min of baseline in the absence (white) or presence (pink) of 78c (100 nM). **(H)** Representative traces from CA/CPR female mice of field excitatory postsynaptic potentials (fEPSPs) before theta burst stimulation (TBS) (gray) and after TBS (pink). LTPexperiments were performed in the same animal in the absence **(left)** and presence of 78c (100 nM) **(right)**. **(I)** Time course graph measuring fEPSPs from CA/CPR female mice normalized to the average ofthe last 10 min of baseline in the absence (white) or presence (pink) of 78c (100 nM). **(J)** Normalized slope of fEPSPs after theta burst stimulus in female mice; *n* = 6–7 animals percondition; Two-Way ANOVA with Repeated Measures, Sidak's *post hoc* test. Values represent mean ± SEM. *p < 0.05; ***p < 0.001.

**Figure 4:**
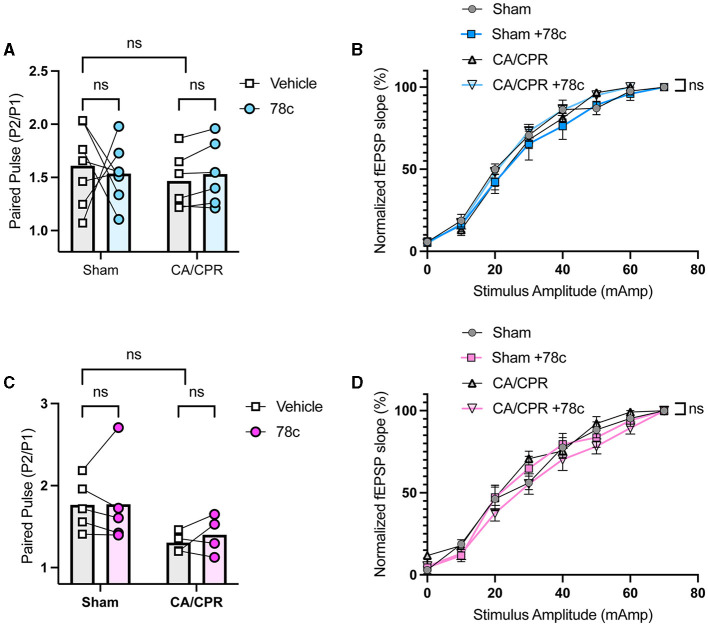
No differences in paired pulse ratio and input-output curve following CD38 inhibition in male and female mice. **(A)** Paired pulse ratio of fEPSP slope, using an interpulse interval of 50 ms in male mice with (blue) or without (white) bath application of 78c; *n* = 5–6 animals per condition; unpaired *t*-test. **(B)** Input-output curve of fEPSP normalized to maximum fEPSP slope in male mice; *n* = 5–6 animals per condition; simple linear regression, slope comparison. **(C)** Paired pulse ratio of fEPSP slope, using an interpulse interval of 50 ms in female mice with (blue) or without (white) bath application of 78c; *n* = 4–5 animals per condition; unpaired *t*-test. **(D)** Input-output curve of fEPSP normalized to maximum fEPSP slope in female mice; *n* = 5–6 animals per condition; simple linear regression, slope comparison.

Our previous study demonstrated ongoing TRPM2 activation contributes to LTP impairments following GCI (Dietz et al., 2020, 2021). Given our hypothesis that CD38 lies upstream of the TRPM2 channel, we next investigated whether CD38 activation in the hippocampus may contribute to hippocampal synaptic plasticity deficits following GCI, phenocopying TRPM2 activation. To address this, we employed field electrophysiology and recorded field excitatory postsynaptic potentials (fEPSPs) from the CA1-Schaffer collateral pathway 7 days following a CA/CPR or sham procedure in both male and female mice. We induced long-term potentiation (LTP), using theta burst stimulation (TBS) and measured LTP in different slices of the same mouse in the absence or presence of 78c (100 nM), a potent CD38 inhibitor, in the bath. We found LTP was intact following the sham procedure with no obvious effect of 78c on the fEPSP waveform (Figure 3A) nor LTP (Sham: 149.6% ± 10.01% vs. Sham+78c: 163.6% ± 13.06% *p* = 0.4740, Figure 3B). Consistent with our previous studies, we found a reduction in LTP following CA/CPR compared to sham (Sham: 149.6% ± 10.01% vs. CA/CPR: 114.8% ± 4.478%, *p* = 0.0430; Figure 3E). In CA/CPR slices treated with 78c, we found a significant restoration of LTP in male mice (CA/CPR: 114.8% ± 4.478% vs. CA/CPR+78c: 151.0% ± 11.10%, *p* = 0.0159; Figures 3C, D) to sham levels (Figure 3E). We obtained similar results in female mice with no effect of the inhibitor on the sham fEPSP waveform (Figure 3F) nor LTP (Sham: 178.8% ± 8.573% vs. Sham+78c: 151.7% ± 10.10%, *p* = 0.0646; Figure 3G). Female CA/CPR exhibited a reduction in LTP compared to sham (Sham: 178.8% ± 8.573% vs. CA/CPR: 118.8% ± 9.198%, *p* = 0.0004; Figure 3J); however, treatment with 78c in CA/CPR hippocampal sections restored LTP (CA/CPR: 118.8% ± 9.198% vs. CA/CPR+78c: 153.9% ± 7.847%, *p* = 0.0204; Figures 3H, I) to sham levels (Figure 3J). We found no differences in release probability between sham and CA/CPR (Sham: 1.609 ± 0.1403 vs. CA/CPR: 1.467 ± 0.1067, *p* = 0.6575) and after 78c application (CA/CPR: 1.467 ± 0.1067 vs. CA/CPR+78c: 1.532 ± 0.1234, *p* = 0.9270) as measured by the paired pulse ratio in both male (Figure 4A) and female mice (Sham: 1.765 ± 0.1390 vs. CA/CPR: 1.306 ± 0.06315, *p* = 0.1434; CA/CPR: 1.306 ± 0.06315 vs. CA/CPR+78c: 1.400 ± 0.1176, *p* = 0.7445; Figure 4C), suggesting primarily postsynaptic mechanism driving these deficits and subsequent recovery with 78c. Further, there were no differences in the slopes of the input-output curves between sham and CA/CPR and following 78c treatment in males (Sham: 1.537 ± 0.1041 vs. CA/CPR: 1.868 ± 0.1319 vs. Sham+78c: 1.586 ± 0.1254 vs. CA/CPR+78c: 1.803 ± 0.09226, *p* = 0.1249; Figure 4B) and females (Sham: 1.563 ± 0.09372 vs. CA/CPR: 1.494 ± 0.1297 vs. Sham+78c: 1.445 ± 0.1253 vs. CA/CPR+78c: 1.4103 ± 0.09402, *p* = 0.7548; Figure 4D), further indicating a postsynaptic mechanism of LTP impairment and restoration by CD38 inhibition that is conserved across sexes.

### CD38 inhibition reverses the OGD-induced enhancement in clustering inhibitory proteins in a TRPM2-dependent manner

**Figure 5:**
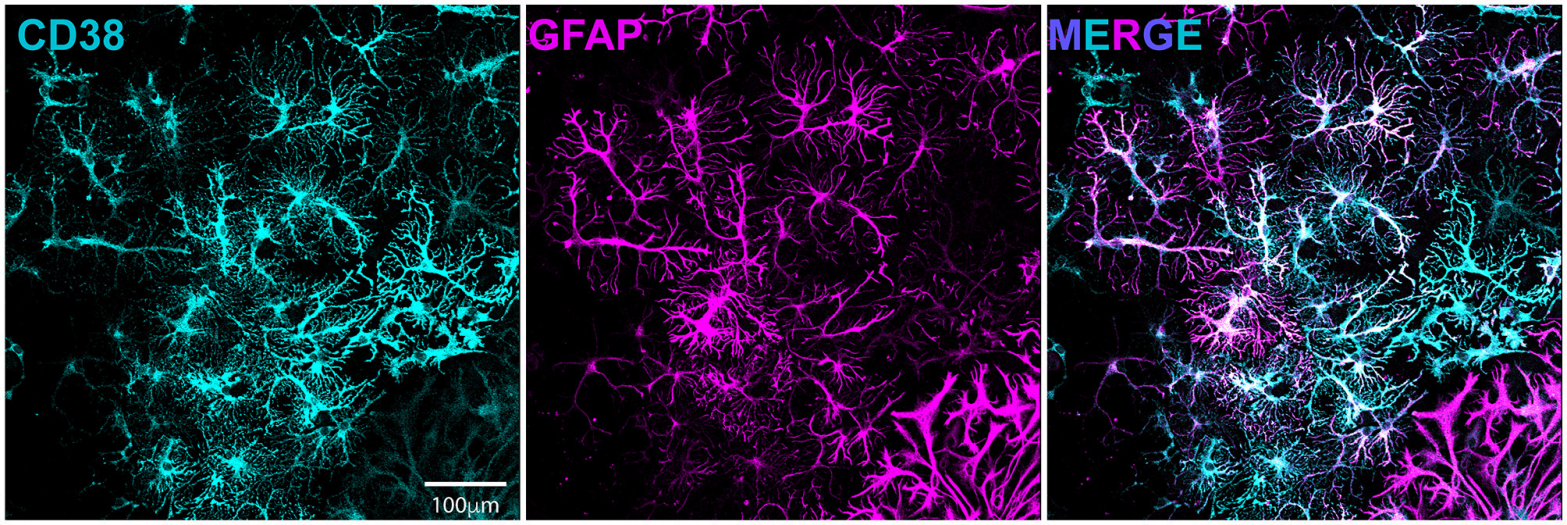
CD38 is expressed in dissociated hippocampal culture and localized to astrocytes. Dissociated hippocampal cultures immunostained for CD38 (cyan, left) and GFAP(magenta, middle).

**Figure 6:**
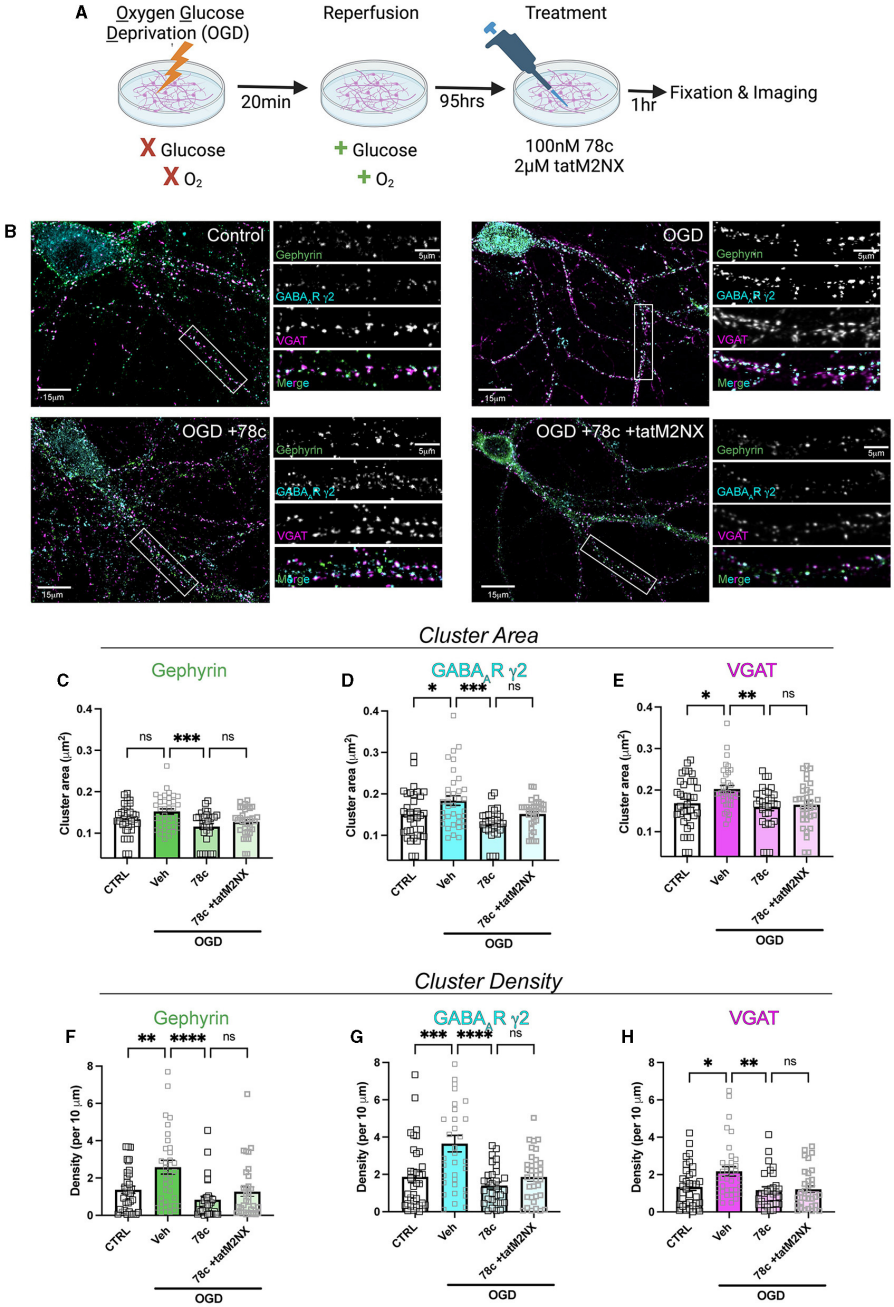
CD38 inhibition reverses the OGD-induced enhancement in clustering of inhibitory proteins in a TRPM2-dependent manner. **(A)** Cartoon illustrating experimental timeline for neurons subjected to OGD-reperfusion andtreated with 78c (100 nM) or tatM2NX (2μM) 1 h prior to fixation. **(B)** Representative confocal images of pyramidal neurons and zoomed in dendritic segmentsimmunostained for gephyrin (green), GABA_A_R-μ2 subunit (cyan), VGAT (magenta). **(C–E)** Quantification of cluster area following treatment with 78c or combined tatM2NX+78c for **(C)** gephyrin, **(D)** surface GABA_A_R-μ2, and **(E)** VGAT; *n* = 30–36 neurons per condition; One-Way ANOVA, Tukey's *post hoc*. **(F–H)** Quantification of cluster density following treatment with 78c or combined tatM2NX+78c for **(F)** gephyrin, **(G)** surface GABA_A_R-μ2, and **(H)** VGAT, *n* = 30–36 neurons per condition;One-Way ANOVA, Tukey's *post hoc*. Values represent mean ± SEM. *p < 0.05; **p < 0.01, ***p < 0.001, ****p < 0.0001.

We previously demonstrated enhanced GABAergic inhibitory synaptic function and clustering likely contributes to the GCI-induced LTP deficits in a TRPM2-dependent manner (Burch et al., 2024). Given our hypothesis that CD38 regulates TRPM2 activation and our previous findings implicating CD38 in GCI-induced LTP impairment (Figure 3), we next investigated whether CD38 may contribute to increased GABA_A_R clustering. To address this, we employed a well-established *in vitro* model of GCI, using an experimental paradigm we have previously shown to induce long-term clustering of GABAergic proteins (Burch et al., 2024). First, we performed immunocytochemistry (ICC) to verify CD38 expression in the cell culture and found robust expression of CD38 in astrocytes labeled with GFAP (Figure 5), consistent to our results obtained *in vivo* (Figure 1). We then subjected mixed-sex, dissociated hippocampal neurons to 20 min of oxygen-glucose deprivation (OGD) and reperfused in conditioned media. After 96 h (4 days), we treated cells with a low concentration of 78c (100 nM), the CD38 inhibitor, or in combination with tatM2NX (2 μM), a potent and specific TRPM2 inhibitor (Cruz-Torres et al., 2020), and fixed 1hr following treatment to assess whether inhibition of CD38 or in combination with TRPM2 restores the clustering of GABAergic proteins at delayed timepoints following ischemia (Figure 6A). We immunostained for gephyrin, the inhibitory scaffolding protein, surface expression of GABA_A_R-μ2 subunit and VGAT, a presynaptic inhibitory marker and measured cluster area and density (Figure 6B). Similar to our previous findings (Burch et al., 2024), we found an increase in the cluster area of surface GABA_A_R-μ2 (CTRL: 0.1492μm^2^ ± 0.009615μm^2^ vs. OGD-Veh: 0.1839μm^2^ ± 0.01138μm^2^, *p* = 0.0285; Figure 6D) and VGAT (CTRL: 0.1686μm^2^ ± 0.009977μm^2^ vs. OGD-Veh: 0.2029μm^2^ ± 0.008316μm^2^, *p* = 0.0378; Figure 6E) in the OGD-vehicle (veh) condition compared to control. Delayed inhibition of CD38 by 78c reduced the OGD-induced increased in cluster area of gephyrin (OGD-Veh: 0.1522μm^2^ ± 0.006524μm^2^ vs. OGD-78c: 0.1162μm^2^ ± 0.007090μm^2^, *p* = 0.0007; Figure 6C), GABA_A_R-μ2 (OGD-Veh: 0.1839μm^2^ ± 0.01138μm^2^ vs. OGD-78c: 0.1282μm^2^ ± 0.006555μm^2^, *p* = 0.0002; Figure 6D), and VGAT (OGD-Veh: 0.2029μm^2^ ± 0.008316μm^2^ vs. OGD-78c: 0.1595μm^2^ ± 0.009128μm^2^, *p* = 0.007; Figure 6E). We found no additive effect on the cluster area of all three GABAergic synaptic markers in the combined tatM2NX and 78c treatment condition (geph, OGD-78c: 0.1162μm^2^ ± 0.007090μm^2^ vs. OGD-78c-tatM2NX: 0.1275μm^2^ ± 0.005773μm^2^, *p* = 0.6152, Figure 6C; GABA_A_R-μ2, OGD-78c: 0.1282μm^2^ ± 0.006555μm^2^ vs. OGD-78c-tatM2NX: 0.1514μm^2^ ± 0.006185μm^2^, *p* = 0.2935, Figure 6D; VGAT, OGD-78c: 0.1595μm^2^ ± 0.009128μm^2^ vs. OGD-78c-tatM2NX: 0.164μm^2^ ± 0.009286μm^2^, *p* = 0.9795, Figure 6E), suggesting CD38-TRPM2 signaling regulate GABAergic synaptic cluster area. We observed similar effects when measuring cluster density. In the veh-OGD condition, we found an increase in the cluster density of gephyrin (CTRL: 1.368 ± 0.1936 vs. OGD-Veh: 2.580 ± 0.3690, *p* = 0.0073; Figure 6F), surface GABA_A_R-μ2 subunit (CTRL: 1.872 ± 0.3060 vs. OGD-Veh: 3.651 ± 0.4383, *p* = 0.0004; Figure 6G) and VGAT (CTRL: 1.345 ± 0.1893 vs. OGD-Veh: 2.176 ± 0.2516, *p* = 0.0213; Figure 6H) compared to control. Treatment with 78c following OGD restored the cluster density of all three markers to control levels (geph, OGD-Veh: 2.580 ± 0.3690 vs. OGD-78c: 0.8334 ± 0.1912, *p* < 0.0001, Figure 6F; GABA_A_R-μ2, OGD-Veh: 3.651 ± 0.4383 vs. OGD-78c: 1.405 ± 0.1853, *p* < 0.0001, Figure 6G; VGAT, OGD-Veh: 2.176 ± 0.2516 vs. OGD-78c: 1.168 ± 0.1818, *p* = 0.0049, Figure 6H), with no additive effect in combination with tatM2NX (geph, OGD-78c: 0.8334 ± 0.1912 vs. OGD-78c-tatM2NX: 1.272 ± 0.2542, *p* = 0.6813, Figure 6F; GABA_A_R-μ2, OGD-78c: 1.405 ± 0.1853 vs. OGD-78c-tatM2NX 1.861 ± 0.2302, *p* = 0.7437, Figure 6G; VGAT, OGD-78c: 1.168 ± 0.1818 vs. OGD-78c-tatM2NX 1.219 ± 0.1834, *p* = 0.9982, Figure 6H). Taken together, this data suggests CD38 and TRPM2 converge on the same pathway to modulate GABAergic synaptic strength, potentially contributing to GCI-induced synaptic plasticity deficits through regulation of inhibitory synapses.

## Discussion

Here, we provide *in vitro* and *in vivo* evidence implicating a CD38-TRPM2 pathway as a potential target to restore hippocampal synaptic plasticity through alterations in GABAergic signaling after GCI. CD38 protein and mRNA expression are increased in activated astrocytes of the CA1-hippocampal region at subacute timepoints following CA/CPR. Ongoing CD38 activation contributes to hippocampal LTP deficits as CD38 inhibition at delayed timepoints restores LTP to sham levels in both sexes, phenocopying TRPM2 inhibition. Further, using an *in vitro* model of brain ischemia, we demonstrated CD38 inhibition reduces clustering of GABAergic synaptic components via a TRPM2-dependent pathway. Our results intriguingly implicate protracted astrogliosis as a driver of perturbed neuronal plasticity in the context of cerebral ischemia and highlight CD38-TRPM2 paracrine signaling as a potential mediator of this process.

Our immunohistochemical and FISH data indicate elevated CD38 expression in activated astrocytes at subacute (7-day timepoints) after GCI. Consistent with this, publicly accessible RNAseq databases indicate preferential CD38 localization to the astrocyte (He et al., 2018; Vanlandewijck et al., 2018). Nevertheless, there is an abundance of literature indicating CD38 is ubiquitously expressed across both peripheral and central immune cell types (Fedele et al., 2004; Amici et al., 2018; Piedra-Quintero et al., 2020; Zambello et al., 2020). Exploring the role of CD38 in neuroimmune cell types such as microglia after ischemia remains an important avenue for future investigation. Despite this caveat, our findings are consistent with prior studies, showing CD38 expression is increased in astrocytes across multiple neuroinflammatory states (Piedra-Quintero et al., 2020). CD38 levels are elevated in patients suffering from HIV-1 encephalitis (Kou et al., 2009). IL-1β stimulation of astrocytes promotes proinflammatory cytokine and chemokine release in a CD38-dependent manner (Mamik et al., 2011). CD38 inhibition in astrocytes reduced glial activation and inflammatory responses cuprizone-induced model of demyelination (Roboon et al., 2019). In the context of ischemia, CD38-deficient mice showed reduced ischemic infarct, neurological deficit and chemokine production; however, its role in astrocytes was unclear due to the use of global CD38 knockout (Choe et al., 2011; Long et al., 2017) Taken together, our data is in agreement with prior studies indicating a role for astroglial CD38 which exacerbates neuroinflammation and is likely detrimental to recovery after a neuroinflammatory insult.

We provide OGD data showing combined CD38 and TRPM2 inhibition has no additive effect in reducing GABAergic clustering, strongly suggests CD38 and TRPM2 converge on the same pathway to regulate neuronal function. Moreover, restoration of GCI-induced LTP impairments by CD38 inhibition (Figure 3), phenocopies TRPM2 reversal of LTP deficits in both sexes. Consistent with this, CD38-TRPM2 signaling has been established in other cellular contexts. Activation of CD38 and TRPM2 regulate oxytocin release in the hypothalamus (Higashida et al., 2018) and CD38-TRPM2-mediated Ca^2+^ signaling are required for hepatic gluconeogenesis (Rah et al., 2023), suggesting this signaling is conserved across multiple cell types. However, it is possible other sources of ADP-ribose are required for TRPM2 activation at the 7-day timepoint studied here. Indeed, in the acute period following ischemic insult, we demonstrated PARP-1 and SIRT-2 generation of ADP-ribose regulates TRPM2 activation and contributes to neuronal injury in male mice (Shimizu et al., 2013, 2016a). However, these enzymes are largely known for their role in oxidative stress-induced cell death pathways (Ha and Snyder, 1999; Zhang et al., 2007; Sarikhani et al., 2018). At the 7-day timepoint studied here, cell death mechanisms are likely completed, making CD38 the most plausible upstream regulator of TRPM2 in this context. Nonetheless, future studies should better resolve the upstream-downstream sequence of this mechanism and more directly investigate the role of CD38-generated ADP-ribose upstream of TRPM2 activation.

In this study, we find no evidence of sexual dimorphism in the increase in astroglial CD38 and the reversal of LTP deficits after CD38 inhibition. This mimics the role of TRPM2 in ischemia-induced synaptic plasticity deficits. Our prior study revealed TRPM2 inhibition at subacute (7 day) and chronic (30 day) timepoints reverses hippocampal-dependent LTP and learning and memory deficits in both male and female mice (Dietz et al., 2020). In contrast, we previously showed TRPM2 genetic depletion or pharmacologic inhibition confers acute neuroprotection in male mice after experimental stroke (Jia et al., 2011; Shimizu et al., 2016b) and GCI (Nakayama et al., 2013). The male-specific activation of TRPM2 in the context of acute neuronal cell death was shown require PARP-1 and SIRT-2, identifying these enzymes as the most likely sources of ADP-ribose and upstream regulators of TRPM2 acutely. Remarkably, our data here suggest CD38-TRPM2 signaling is distinct to the subacute and chronic phases, mediating synaptic dysfunction of the surviving neuronal network that is conserved across both sexes. Targeting this pathway would therefore broaden its therapeutic relevance, highlighting its potential efficacy in both males and females.

In summary, we propose a novel CD38-TRPM2 mechanism, linking protracted astrogliosis to neuronal dysfunction at delayed timepoints following GCI. Enhanced expression of astroglial CD38 in the hippocampus correlates with LTP deficits following GCI through augmentation of GABA_A_R clustering. Targeting this novel pathologic paracrine signaling offers strong therapeutic potential to reverse hippocampal-dependent cognitive impairment weeks to months after initial ischemic insult.

## Data availability statement

The original contributions presented in the study are included in the article/Supplementary material, further inquiries can be directed to the corresponding author.

## Ethics statement

The animal study was approved by the University of Colorado Anschutz Medical Campus. The study was conducted in accordance with the local legislation and institutional requirements.

## Author contributions

AB: Conceptualization, Data curation, Formal analysis, Investigation, Methodology, Writing – original draft, Writing – review & editing. AH: Investigation, Methodology, Writing – review & editing. JO: Data curation, Investigation, Writing – review & editing. ET: Investigation, Writing – review & editing. CG: Investigation, Writing – review & editing. NC: Data curation, Formal analysis, Investigation, Writing – review & editing. NQ: Conceptualization, Formal analysis, Investigation, Resources, Supervision, Writing – original draft, Writing – review & editing. PH: Conceptualization, Funding acquisition, Project administration, Resources, Supervision, Writing – original draft, Writing – review & editing.

## References

[B1] Amici S. A. Young N. A. Narvaez-Miranda J. Jablonski K. A. Arcos J. Rosas L.. (2018). CD38 is robustly induced in human macrophages and monocytes in inflammatory conditions. Front. Immunol. 9:593. 10.3389/fimmu.2018.01593 30042766 PMC6048227

[B2] Azad T. D. Veeravagu A. Steinberg G. K. (2016). Neurorestoration after stroke. Neurosurg. Focus 40:E2. 10.3171/2016.2.FOCUS15637 27132523 PMC4916840

[B3] Burch A. M. Garcia J. D. O'Leary H. Haas A. Orfila J. E. Tiemeier E.. (2024). TRPM2 and CaMKII signaling drives excessive GABAergic synaptic inhibition following ischemia. J. Neurosci. 44:e1762232024. 10.1523/JNEUROSCI.1762-23.2024 38565288 PMC11079974

[B4] Chatterjee S. Daenthanasanmak A. Chakraborty P. Wyatt M. W. Dhar P. Selvam S. P.. (2018). CD38-NAD(+)axis regulates immunotherapeutic anti-tumor T cell response. Cell Metab. 27, 85–100.e108. 10.1016/j.cmet.2017.10.006 29129787 PMC5837048

[B5] Cheng Y. D. Al-Khoury L. Zivin J. A. (2004). Neuroprotection for ischemic stroke: two decades of success and failure. NeuroRx 1, 36–45. 10.1602/neurorx.1.1.36 15717006 PMC534911

[B6] Chini C. C. Peclat T. R. Warner G. M. Kashyap S. Espindola-Netto J. M. de Oliveira G. C.. (2020). CD38 ecto-enzyme in immune cells is induced during aging and regulates NAD(+) and NMN levels. Nat Metab 2, 1284–1304. 10.1038/s42255-020-00298-z 33199925 PMC8752031

[B7] Choe C. U. Lardong K. Gelderblom M. Ludewig P. Leypoldt F. Koch-Nolte F.. (2011). CD38 exacerbates focal cytokine production, postischemic inflammation and brain injury after focal cerebral ischemia. PLoS ONE 6:e19046. 10.1371/journal.pone.0019046 21625615 PMC3097994

[B8] Crosby K. C. Gookin S. E. Garcia J. D. Hahm K. M. Dell'Acqua M. L. Smith K. R. (2019). Nanoscale subsynaptic domains underlie the organization of the inhibitory synapse. Cell Rep. 26, 3284–3297.e3283. 10.1016/j.celrep.2019.02.070 30893601 PMC6529211

[B9] Cruz-Torres I. Backos D. S. Herson P. S. (2020). Characterization and Optimization of the Novel Transient Receptor Potential Melastatin 2 Antagonist tatM2NX. Mol. Pharmacol. 97, 102–111. 10.1124/mol.119.117549 31772034 PMC6964147

[B10] Deng G. Orfila J. E. Dietz R. M. Moreno-Garcia M. Rodgers K. M. Coultrap S. J.. (2017). Autonomous CaMKII activity as a drug target for histological and functional neuroprotection after resuscitation from cardiac arrest. Cell Rep. 18, 1109–1117. 10.1016/j.celrep.2017.01.011 28147268 PMC5540152

[B11] Dietz R. M. Cruz-Torres I. Orfila J. E. Patsos O. P. Shimizu K. Chalmers N.. (2020). Reversal of global ischemia-induced cognitive dysfunction by delayed inhibition of TRPM2 ion channels. Transl. Stroke Res. 11, 254–266. 10.1007/s12975-019-00712-z 31250378 PMC6934940

[B12] Dietz R. M. Orfila J. E. Chalmers N. Minjarez C. Vigil J. Deng G.. (2021). Functional restoration following global cerebral ischemia in juvenile mice following inhibition of transient receptor potential M2 (TRPM2) ion channels. Neural Plast. 2021:8774663. 10.1155/2021/8774663 34659399 PMC8514917

[B13] Escobar I. Xu J. Jackson C. W. Perez-Pinzon M. A. (2019). Altered neural networks in the papez circuit: implications for cognitive dysfunction after cerebral ischemia. J. Alzheimers. Dis. 67, 425–446. 10.3233/JAD-180875 30584147 PMC6398564

[B14] Fedele G. Frasca L. Palazzo R. Ferrero E. Malavasi F. Ausiello C. M. (2004). CD38 is expressed on human mature monocyte-derived dendritic cells and is functionally involved in CD83 expression and IL-12 induction. Eur. J. Immunol. 34, 1342–1350. 10.1002/eji.200324728 15114667

[B15] Garcia J. D. Gookin S. E. Crosby K. C. Schwartz S. L. Tiemeier E. Kennedy M. J.. (2021). Stepwise disassembly of GABAergic synapses during pathogenic excitotoxicity. Cell Rep. 37:110142. 10.1016/j.celrep.2021.110142 34936876 PMC8824488

[B16] Ha H. C. Snyder S. H. (1999). Poly(ADP-ribose) polymerase is a mediator of necrotic cell death by ATP depletion. Proc. Natl. Acad. Sci. USA. 96, 13978–13982. 10.1073/pnas.96.24.13978 10570184 PMC24176

[B17] Hannawi Y. Ewees M. G. Moore J. T. Zweier J. L. (2022). Characterizing CD38 expression and enzymatic activity in the brain of spontaneously hypertensive stroke-prone rats. Front. Pharmacol. 13:881708. 10.3389/fphar.2022.881708 35712720 PMC9194821

[B18] He L. Vanlandewijck M. Mäe M. A. Andrae J. Ando K. Del Gaudio F.. (2018). Single-cell RNA sequencing of mouse brain and lung vascular and vessel-associated cell types. Sci Data 5:180160. 10.1038/sdata.2018.160 30129931 PMC6103262

[B19] Higashida H. Yuhi T. Akther S. Amina S. Zhong J. Liang M.. (2018). Oxytocin release via activation of TRPM2 and CD38 in the hypothalamus during hyperthermia in mice: Implication for autism spectrum disorder. Neurochem. Int. 119, 42–48. 10.1016/j.neuint.2017.07.009 28736241

[B20] Hogan K. A. Chini C. C. S. Chini E. N. (2019). The multi-faceted ecto-enzyme CD38: roles in immunomodulation, cancer, aging, and metabolic diseases. Front. Immunol. 10:1187. 10.3389/fimmu.2019.01187 31214171 PMC6555258

[B21] Jia J. Verma S. Nakayama S. Quillinan N. Grafe M. R. Hurn P. D.. (2011). Sex differences in neuroprotection provided by inhibition of TRPM2 channels following experimental stroke. J. Cereb. Blood Flow Metab. 31, 2160–2168. 10.1038/jcbfm.2011.77 21587268 PMC3210342

[B22] Katz A. Brosnahan S. B. Papadopoulos J. Parnia S. Lam J. Q. (2022). Pharmacologic neuroprotection in ischemic brain injury after cardiac arrest. Ann. N. Y. Acad. Sci. 1507, 49–59. 10.1111/nyas.14613 34060087

[B23] Kou W. Banerjee S. Eudy J. Smith L. M. Persidsky R. Borgmann K.. (2009). CD38 regulation in activated astrocytes: implications for neuroinflammation and HIV-1 brain infection. J. Neurosci. Res. 87, 2326–2339. 10.1002/jnr.22060 19365854

[B24] Leao R. N. Mikulovic S. Leao K. E. Munguba H. Gezelius H. Enjin A.. (2012). OLM interneurons differentially modulate CA3 and entorhinal inputs to hippocampal CA1 neurons. Nat. Neurosci. 15, 1524–1530. 10.1038/nn.3235 23042082 PMC3483451

[B25] Long A. Park J. H. Klimova N. Fowler C. Loane D. J. Kristian T. (2017). CD38 knockout mice show significant protection against ischemic brain damage despite high level poly-ADP-ribosylation. Neurochem. Res. 42, 283–293. 10.1007/s11064-016-2031-9 27518087 PMC5580240

[B26] Luscher B. Fuchs T. Kilpatrick C. L. (2011). GABAA receptor trafficking-mediated plasticity of inhibitory synapses. Neuron 70, 385–409. 10.1016/j.neuron.2011.03.024 21555068 PMC3093971

[B27] Malavasi F. Deaglio S. Funaro A. Ferrero E. Horenstein A. L. Ortolan E.. (2008). Evolution and function of the ADP ribosyl cyclase/CD38 gene family in physiology and pathology. Physiol. Rev. 88, 841–886. 10.1152/physrev.00035.2007 18626062

[B28] Mamik M. K. Banerjee S. Walseth T. F. Hirte R. Tang L. Borgmann K.. (2011). HIV-1 and IL-1beta regulate astrocytic CD38 through mitogen-activated protein kinases and nuclear factor-kappaB signaling mechanisms. J. Neuroinflam. 8:145. 10.1186/1742-2094-8-145 22027397 PMC3247131

[B29] Mayo L. Jacob-Hirsch J. Amariglio N. Rechavi G. Moutin M. J. Lund F. E.. (2008). Dual role of CD38 in microglial activation and activation-induced cell death. J. Immunol. 181, 92–103. 10.4049/jimmunol.181.1.92 18566373 PMC3683558

[B30] Moulaert V. R. Verbunt J. A. van Heugten C. M. Wade D. T. (2009). Cognitive impairments in survivors of out-of-hospital cardiac arrest: a systematic review. Resuscitation 80, 297–305. 10.1016/j.resuscitation.2008.10.034 19117659

[B31] Nakayama S. Vest R. Traystman R. J. Herson P. S. (2013). Sexually dimorphic response of TRPM2 inhibition following cardiac arrest-induced global cerebral ischemia in mice. J. Mol. Neurosci. 51, 92–98. 10.1007/s12031-013-0005-9 23532768 PMC3728170

[B32] Nusser Z. Cull-Candy S. Farrant M. (1997). Differences in synaptic GABA(A) receptor number underlie variation in GABA mini amplitude. Neuron 19, 697–709. 10.1016/S0896-6273(00)80382-7 9331359

[B33] Orfila J. E. Shimizu K. Garske A. K. Deng G. Maylie J. Traystman R. J.. (2014). Increasing small conductance Ca2+-activated potassium channel activity reverses ischemia-induced impairment of long-term potentiation. Eur. J. Neurosci. 40, 3179–3188. 10.1111/ejn.12683 25080203 PMC4205199

[B34] Pekny M. Nilsson M. (2005). Astrocyte activation and reactive gliosis. Glia 50, 427–434. 10.1002/glia.20207 15846805

[B35] Pekny M. Wilhelmsson U. Pekna M. (2014). The dual role of astrocyte activation and reactive gliosis. Neurosci. Lett. 565, 30–38. 10.1016/j.neulet.2013.12.071 24406153

[B36] Pekny M. Wilhelmsson U. Tatlisumak T. Pekna M. (2019). Astrocyte activation and reactive gliosis-A new target in stroke? Neurosci. Lett. 689, 45–55. 10.1016/j.neulet.2018.07.021 30025833

[B37] Petito C. K. Feldmann E. Pulsinelli W. A. Plum F. (1987). Delayed hippocampal damage in humans following cardiorespiratory arrest. Neurology 37, 1281–1286. 10.1212/WNL.37.8.1281 3614648

[B38] Piedra-Quintero Z. L. Wilson Z. Nava P. Guerau-de-Arellano M. (2020). CD38: An Immunomodulatory Molecule in Inflammation and Autoimmunity. Front. Immunol. 11:597959. 10.3389/fimmu.2020.597959 33329591 PMC7734206

[B39] Rah S. Y. Joe Y. Park J. Ryter S. W. Park C. Chung H. T.. (2023). CD38/ADP-ribose/TRPM2-mediated nuclear Ca(2+) signaling is essential for hepatic gluconeogenesis in fasting and diabetes. Exp. Mol. Med. 55, 1492–1505. 10.1038/s12276-023-01034-9 37394593 PMC10393965

[B40] Rajgor D. Purkey A. M. Sanderson J. L. Welle T. M. Garcia J. D. Dell'Acqua M. L.. (2020). Local miRNA-dependent translational control of GABA(A)R synthesis during inhibitory long-term potentiation. Cell Rep. 31:107785. 10.1016/j.celrep.2020.107785 32579917 PMC7486624

[B41] Roboon J. Hattori T. Ishii H. Takarada-Iemata M. Le T. M. Shiraishi Y.. (2019). Deletion of CD38 suppresses glial activation and neuroinflammation in a mouse model of demyelination. Front. Cell. Neurosci. 13:258. 10.3389/fncel.2019.00258 31244614 PMC6563778

[B42] Sabedra A. R. Kristan J. Raina K. Holm M. B. Callaway C. W. Guyette F. X.. (2015). Neurocognitive outcomes following successful resuscitation from cardiac arrest. Resuscitation 90, 67–72. 10.1016/j.resuscitation.2015.02.023 25737082 PMC4404201

[B43] Sarikhani M. Mishra S. Desingu P. A. Kotyada C. Wolfgeher D. Gupta M. P.. (2018). SIRT2 regulates oxidative stress-induced cell death through deacetylation of c-Jun NH(2)-terminal kinase. Cell Death Differ. 25, 1638–1656. 10.1038/s41418-018-0069-8 29449643 PMC6143545

[B44] Shimizu K. Quillinan N. Orfila J. E. Herson P. S. (2016a). Sirtuin-2 mediates male specific neuronal injury following experimental cardiac arrest through activation of TRPM2 ion channels. Exp. Neurol. 275, 78–83. 10.1016/j.expneurol.2015.10.014 26522013 PMC5193101

[B45] Shimizu T. Dietz R. M. Cruz-Torres I. Strnad F. Garske A. K. Moreno M.. (2016b). Extended therapeutic window of a novel peptide inhibitor of TRPM2 channels following focal cerebral ischemia. Exp. Neurol. 283, 151–156. 10.1016/j.expneurol.2016.06.015 27317297 PMC5240152

[B46] Shimizu T. Macey T. A. Quillinan N. Klawitter J. Perraud A. L. Traystman R. J.. (2013). Androgen and PARP-1 regulation of TRPM2 channels after ischemic injury. J. Cereb. Blood Flow Metab. 33, 1549–1555. 10.1038/jcbfm.2013.105 23801245 PMC3790922

[B47] Smith K. R. Kittler J. T. (2010). The cell biology of synaptic inhibition in health and disease. Curr. Opin. Neurobiol. 20, 550–556. 10.1016/j.conb.2010.06.001 20650630

[B48] Steele P. M. Mauk M. D. (1999). Inhibitory control of LTP and LTD: stability of synapse strength. J. Neurophysiol. 81, 1559–1566. 10.1152/jn.1999.81.4.1559 10200191

[B49] Tarrago M. G. Chini C. C. S. Kanamori K. S. Warner G. M. Caride A. de Oliveira G. C.. (2018). A potent and specific CD38 inhibitor ameliorates age-related metabolic dysfunction by reversing tissue NAD(+) decline. Cell Metab. 27, 1081–1095.e1010. 10.1016/j.cmet.2018.03.016 29719225 PMC5935140

[B50] Udakis M. Pedrosa V. Chamberlain S. E. L. Clopath C. Mellor J. R. (2020). Interneuron-specific plasticity at parvalbumin and somatostatin inhibitory synapses onto CA1 pyramidal neurons shapes hippocampal output. Nat. Commun. 11:4395. 10.1038/s41467-020-18074-8 32879322 PMC7467931

[B51] Vanlandewijck M. He L. Mae M. A. Andrae J. Ando K. Del Gaudio F.. (2018). A molecular atlas of cell types and zonation in the brain vasculature. Nature 554, 475–480. 10.1038/nature25739 29443965

[B52] Vogels T. P. Sprekeler H. Zenke F. Clopath C. Gerstner W. (2011). Inhibitory plasticity balances excitation and inhibition in sensory pathways and memory networks. Science 334, 1569–1573. 10.1126/science.1211095 22075724

[B53] Wahlgren N. G. Ahmed N. (2004). Neuroprotection in cerebral ischaemia: facts and fancies–the need for new approaches. Cerebrovasc. Dis. 17, 153–166. 10.1159/000074808 14694293

[B54] Williams L. E. Holtmaat A. (2019). Higher-Order Thalamocortical Inputs Gate Synaptic Long-Term Potentiation via Disinhibition. Neuron 101, 91–102.e104. 10.1016/j.neuron.2018.10.049 30472077

[B55] Zambello R. Barila G. Manni S. Piazza F. Semenzato G. (2020). NK cells and CD38: implication for (immuno)therapy in plasma cell dyscrasias. Cells 9:768. 10.3390/cells9030768 32245149 PMC7140687

[B56] Zhang S. Lin Y. Kim Y. S. Hande M. P. Liu Z. G. Shen H. M. (2007). c-Jun N-terminal kinase mediates hydrogen peroxide-induced cell death via sustained poly(ADP-ribose) polymerase-1 activation. Cell Death Differ. 14, 1001–1010. 10.1038/sj.cdd.4402088 17218956

