# Supplementary material for: Astroglial CD38 impairs hippocampal synaptic plasticity after global cerebral ischemia
Source: Front Stroke. 2024 Aug 14;3:1423887. doi: 10.3389/fstro.2024.1423887 (PMC12356413; doi:10.3389/fstro.2024.1423887)
Supplement: Supplementary file 1 [file Data_Sheet_1.DOCX]

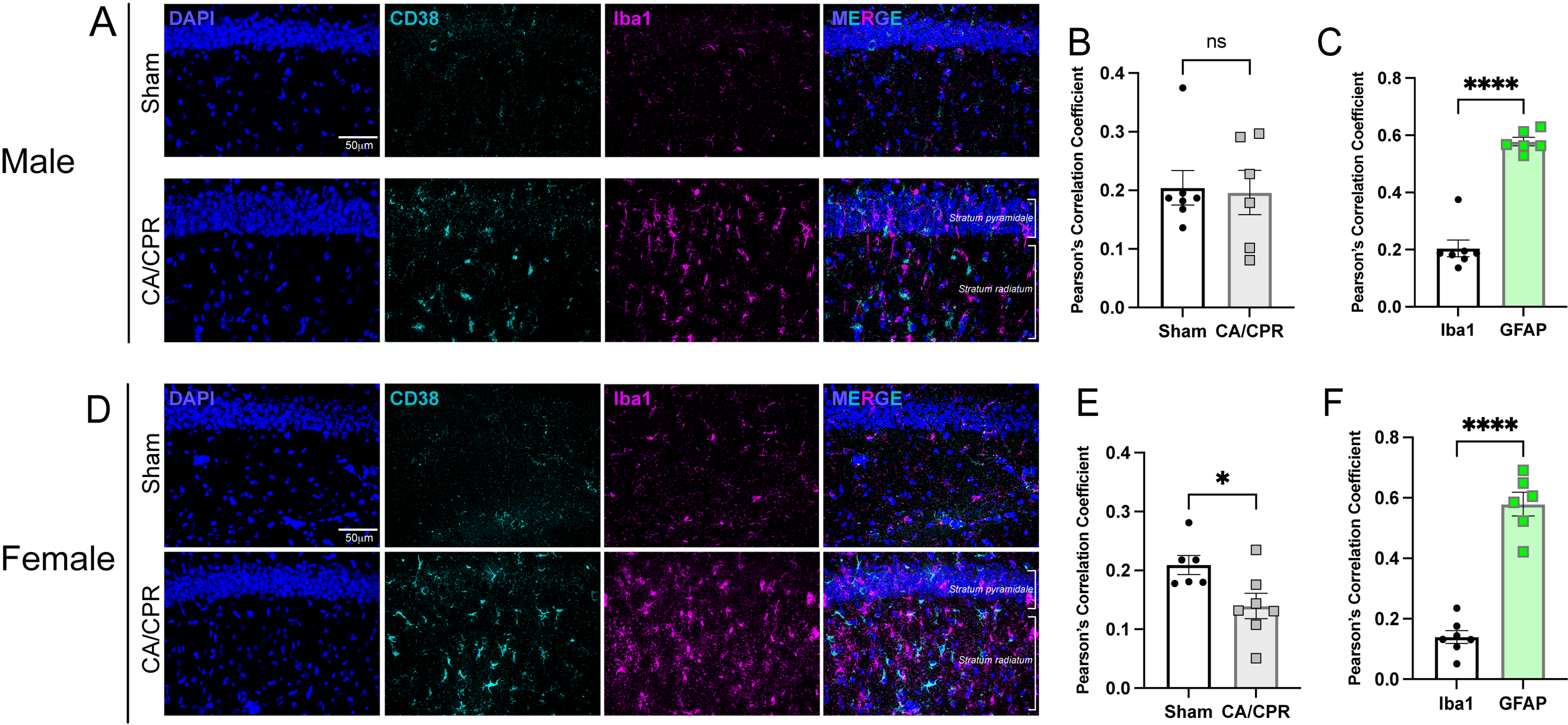


**Figure S1. CD38 is not colocalized to microglia after CA/CPR in both male and female mice.**

A. Representative confocal images of DAPI (blue), CD38 (cyan) and Iba1 (magenta) immunohistochemical staining from CA1 hippocampal sections of 7-day sham and CA/CPR male mice.

B. Quantification of CD38 colocalization to Iba1 using Pearson’s Correlation Coefficient in male mice; n=6 animals per condition; unpaired t-test.

C. Quantification of CD38 colocalization to Iba1 compared to GFAP after CA/CPR in male mice; n=6 animals per condition; unpaired t-test.

D. Representative confocal images of DAPI (blue), CD38 (cyan) and Iba1 (magenta) immunohistochemical staining from CA1 hippocampal sections of 7-day sham and CA/CPR female mice.

E. Quantification of CD38 colocalization to Iba1 using Pearson’s Correlation Coefficient in female mice; n=6-7 animals per condition; unpaired t-test.

F. Quantification of CD38 colocalization to Iba1 compared to GFAP after CA/CPR in female mice; n=6-7 animals per condition; unpaired t-test.

Values represent mean ± SEM. *p<0.05; ** p<0.01, ***p<0.001.
